# Tetracyclines—An Important Therapeutic Tool for Dermatologists

**DOI:** 10.3390/ijerph19127246

**Published:** 2022-06-13

**Authors:** Malgorzata Orylska-Ratynska, Waldemar Placek, Agnieszka Owczarczyk-Saczonek

**Affiliations:** Department of Dermatology, Sexually Transmitted Diseases and Clinical Immunology, University of Warmia and Mazury in Olsztyn, Oczapowskiego 2, 10-719 Olsztyn, Poland; w.placek@wp.pl (W.P.); aganek@wp.pl (A.O.-S.)

**Keywords:** tetracyclines, doxycycline, limecycline, minocycline, pleiotrophy, non-antibiotic properties

## Abstract

Tetracyclines are a group of antibiotics whose first representative was discovered over 70 years ago. Since then, they have been of great interest in dermatology. In addition to their antibacterial activity, they are able to inhibit metalloproteinases and exhibit anti-inflammatory, anti-apoptotic and antioxidant effects. The side effects have been thoroughly studied over the years, the most characteristic and important ones in daily dermatological practice being: phototoxicity, hyperpigmentation, onycholysis, photoonycholysis, induced lupus erythematosus, and idiopathic intracranial hypertension. In this article, we summarize the use of tetracyclines in infectious diseases and inflammatory dermatoses, and further discuss the instances where the efficacy and safety of tetracyclines have been highlighted over the past few years.

## 1. Introduction

Tetracyclines are natural compounds produced by Streptomyces species that were discovered by Benjamin Duggar in 1948. Today, we have a whole class of drugs derived from the substance discovered then.

The product of soil bacteria from the Streptomyces family was quickly subjected to careful analysis and subsequent modification. Tetracyclines began to be obtained cheaply and rapidly by fermentation [1]. This contributed to their widespread use in the treatment of human and animal diseases, animal growth promotion and aquaculture [2,3] (see Table 1).

The first, and best characterized, representative of the group was chlortetracycline [7]. Compared with the penicillins already available at that time, chlortetracycline also had an effect on Gram-negative bacteria and was better tolerated by patients. Soon afterwards, other natural tetracyclines were isolated, including tetracycline, from which this family of molecules takes its name.

The modifications of naturally occurring tetracyclines and the synthesis of new compounds resulted in the development of new antibiotics in this group.

The development of chemically modified tetracyclines (CMT) was the most driven by common side effects of older-generation tetracyclines, e.g., gastrointestinal disorders, developmental bone and tooth deformities and the development of resistant bacterial strains [8].

The tetracycline group includes tetracycline, doxycycline, minocycline, lymecycline and sarecycline.

Chemically, the tetracyclic naphthacene-carboxamide ring system is the basic structure of all drugs in this group. The dimethylamine group at the C4 carbon is responsible for the antibacterial properties of tetracyclines. The removal of the side chain reduces antibacterial properties and enhances non-antibiotic activity, and was used in the production of second-generation tetracyclines [4].

### Mechanism of Antibacterial Action

Tetracyclines passively diffuse through pores in the bacterial membrane [9]. The bacteriostatic potential of tetracyclines is based on ribosome inactivation. When bound to the 30S subunit, tetracyclines inhibit protein biosynthesis, causing bacterial death.

The biological activity of tetracyclines is strongly dependent on metal chelation. In circulation, the non-protein-bound fraction of tetracyclines mainly acts as ionophores for divalent metals. Ionophores are compounds capable of forming lipid-soluble complexes with metal cations [10,11].

The lower half of the tetracycline molecule is a key region for binding metal ions. Therefore, it influences the binding of bacterial ribosome, metalloproteinase-family enzymes, and mediates the mechanism of resistance [5,12].

Calcium (Ca^2+^) is the main cation found in the plasma, while magnesium (Mg^2+^) is found in the intercellular space [13,14,15].

In the case of tetracycline, chelation also affects drug absorption in the gastrointestinal tract. Taking the drug with a meal rich in calcium leads to the formation of insoluble compounds and disruption in drug absorption. It is necessary to maintain an interval between tetracycline administration and a meal, i.e., at least 1 h before or 2 h after a meal [16]. The higher lipophilicity of the subsequent generations of tetracyclines provides better absorption and a lower predisposition to the formation of chelates with metal ions.

Tetracyclines (TCs) form a stable complex with calcium in any bone-forming tissue, tending to deposit in the areas of calcification in bones and teeth. This accumulation may be associated with adverse effects, such as permanent tooth pigmentation, especially in young individuals [17].

The extensive use of tetracyclines over the past four decades has led to the development of bacterial resistance.

The most common mechanism of the resistance of Gram-negative bacteria is associated with the membrane protein TetA, which exports the drug from bacterial cells before it manages to attack its target, i.e., ribosomes [18].

The phenomenon of chelation is also of key significance in the mechanism of resistance. After binding tetracycline to a metal inside bacteria, the membrane protein starts to act as an antiporter removing the tetracycline–metal complex in exchange for the inflow of a proton [19].

As regards clinicians, the largest quantities of antibiotics are prescribed by dermatologists, with tetracyclines constituting about 75% [20].

Due to increasing bacterial resistance, tetracyclines lost their significance as antimicrobial drugs, but they are still extensively used due to their numerous non-antibiotic properties.

## 2. Non-Antibiotic Properties of Tetracyclines

### 2.1. Inhibiting Metalloproteinases

The matrix metalloproteinases (MMP) are a family of zinc-dependent proteases produced by inflammatory cells and connective tissue cells which participate in numerous physiological and pathophysiological processes [21].

An increased activity of metalloproteinases is observed in almost all human conditions associated with inflammation [22].

Tetracyclines inhibit the activity of metalloproteinases binding an ion of zinc or calcium included in the enzyme structure [6].

Three main classes of metalloproteinases are distinguished: collagenases, gelatinases and stromelysins. The majority of data regarding the influence of tetracyclines on metalloproteinases are derived from research on their use in periodontal diseases. Polymorphonuclear leukocytes (PMNs) are the main source of collagenases. They mediate connective tissue breakdown during inflammatory periodontal disease, while fibroblasts provide the collagenase necessary for connective tissue remodeling in the normal gingiva. It was demonstrated that tetracyclines did not impair the production of the enzyme by fibroblasts to ensure appropriate collagen turnover. They only influenced collagenases produced by neutrophils [23].

In addition to metalloproteinases, tetracyclines also inhibit enzymes from the hydrolase group alpha-amylases and phospholipases. Phospholipase A2 is a key enzyme in the biosynthesis of inflammatory mediators such as prostaglandins [24].

### 2.2. Anti-Inflammatory Activity

The broadly understood anti-inflammatory effect of tetracyclines was first observed in rosacea. The pathogenesis of rosacea has long excluded the direct involvement of bacterial factors, and yet the effectiveness of tetracyclines was observed. Today, there is ample evidence that tetracyclines reduce the inflammation of various etiologies [25].

Doses below the minimal inhibitory concentration indirectly affect inflammation by inhibiting bacterial breakdown products, which stimulate inflammatory processes.

They reduce the production of pro-inflammatory cytokines by neutrophils, such as interleukin-1β (IL-1β), IL-8, and tumor necrosis factor-α [26].

They inhibit leukocyte migration, which occurs during the early stages of inflammation. Cell movement is inhibited by binding intracellular calcium, which is necessary for the formation of microtubules that enable cell movement [27].

Tetracyclines reduce nitric oxide (NO) by inhibiting inducible nitric oxide synthase (iNOS) activity. The final product of iNOS is a highly cytotoxic peroxynitrite radical responsible for inhibiting collagen synthesis and proteoglycans, and increasing MMP expression. Tetracyclines prevent protein denaturation by reducing peroxynitrite radicals [28].

NO likely mediates increased vessel permeability and edema and may support erythema development concomitant with rosacea.

### 2.3. Antioxidant Effect

Tetracyclines also scavenge reactive oxygen species (ROS) and prevent oxidative damage to cell structures [29].

The phenolic ring is crucial for the ability of these compounds to retain ROS. Upon attachment of a free radical to the phenolic ring, a stable phenolic radical is formed which, surrounded by the side groups of the phenolic ring, does not undergo further interactions [30].

### 2.4. Anti-Apoptotic Effect

Apoptosis is a tightly regulated program of cell death. The initiating phenomenon of apoptosis involves the activation of caspases, which follows a hierarchical pattern in which higher-order caspases are activated by initial apoptotic signals and then cleave and activate lower-order caspases, which then carry out proteolysis [31]. Two pathways can lead to caspase activation.

The extrinsic pathway is triggered by the binding of death signal proteins to a receptor on the cell surface. This binding activates caspase-8, which then activates effector caspases.

The intrinsic pathway can be initiated by both intracellular and extracellular stimuli. This leads to an increase in mitochondrial membrane permeability and the release of cytochrome c into the cytoplasm. The formation of the apoptosome (a multiprotein composite formed by cytochrome c) activates caspase-9, initiating further caspase activation [29,32].

Also taking place in the intrinsic pathway via the mitochondrial regulation inhibitor of SMAC and DIABLO, groups of proteins bind to the inhibitor of apoptosis proteins (IAP) and by inactivating them allow apoptosis to proceed. Another group of proteins, called apoptosis-inducing factors (AIF), function in a caspase-independent pathway and cause DNA fragmentation inside the cell nucleus [33,34,35,36].

In vitro studies on animal models showed that tetracyclines caused a decrease in caspase expression [37]. The accumulation of tetracyclines in mitochondria was also demonstrated, as well as the possibility of altering the membrane potential, which interferes with the synthesis of proteins encoded by mitochondrial DNA [32,38]. The inhibition of cytochrome c release is another proposed mechanism [32].

The most information on the anti-apoptotic properties of tetracyclines was obtained from studies of the properties of minocycline in psychiatric and neurodegenerative diseases.

A total of 83% of all selected studies conducted on animal models presented positive results. However, this percentage was much lower in randomized clinical trials, mainly due to the different doses of the drug used in both types of studies and the difficulty in selecting patients at the same stage of the disease. Furthermore, some of the symptoms of these diseases are impossible to reproduce in an animal model [39].

Such encouraging results from preclinical studies require further standardization and the improvement of clinical protocols in order to objectify the results.

## 3. Types of Tetracyclines

### 3.1. Tetracycline

Tetracycline is the first representative of the group. It is formed of four six-carbon rings with attached methyl and hydroxyl groups at the C6 carbon [40].

After oral administration it is rapidly absorbed from the digestive tract, with the maximal concentration being reached after 1–3 h. However, the absorption may be reduced in the presence of milk products or preparations including metal ions.

### 3.2. Doxycycline

Doxycycline consists of four identical six-carbon rings with an additional hydroxyl group at the C5 carbon and a methyl group at the C6 carbon [41].

It was approved by the FDA in the treatment of severe forms of acne at the dose of 50–100 mg 1–2 times daily [42].

As regards doxycycline, the effectiveness of the dose, not including the bacteriostatic activity, was precisely demonstrated, both in acne vulgaris and in acne rosacea.

In Europe and the United States, doxycycline with exclusive anti-inflammatory activity is available at the dose of 40 mg, which includes 30 mg of immediate-release monohydrate and 10 mg in slow-release microgranule form. The preparation is entirely devoid of antibacterial activity. In 2006 it was approved by the FDA for the treatment of acne rosacea [43].

Doxycycline at a dose of 20 mg was registered for the treatment of periodontal diseases in adults, and its efficacy in this indication was ascribed to the inhibiting activity of collagenase and matrix metalloproteinase [44,45].

Doxycycline is the first-line treatment in wide range of infections including *Treponema pallidum* (syphilis), *Borrelia burgdorferi, B. afzelii, B. garinii* (borreliosis), *Coxiella burnetii* (Q fever), *Rickettsia rickettsii* (Rocky Mountain spotted fever) and *Yersinia pestis* (plague) [46].

### 3.3. Lymecycline

Lymecycline is a semisynthetic tetracycline, developed by combining tetracycline with L-lysine. Compared with tetracycline it is characterized by higher absorption levels, enhanced tissue penetration, higher serum levels, and slower elimination [47].

Due to the highest oral absorption of all tetracyclines [48], lymecycline is administered as a simple, once-daily regimen. It induces fewer adverse effects than previously used drugs from this group [49].

It is unavailable in North America.

A multicenter, randomized blinded study conducted to compare the efficacy, safety and cost-effectiveness of using lymecycline and minocycline revealed that both drugs had a similar safety and efficacy profile, while the cost of treatment with lymecycline was 4-fold lower [50,51,52].

### 3.4. Minocycline

Minocycline consists of a tetracyclic ring with an additional dimethyl amino group at the C7 carbon which makes it the most lipophilic tetracycline.

Due to this property, it crosses the blood–brain barrier and reaches high concentrations in the central nervous system, which explains more frequent occurrence of adverse effects such as nausea, vomiting and vertigo [53].

It was approved by the FDA in the treatment of severe acne at a dose of 50–135 mg/day.

When treatment is continued for more than 6 months it may cause characteristic pigmentation, lupus-like lesions and irritable bowel syndrome [22,54].

### 3.5. Sarecycline

Sarecycline is a new narrow-spectrum antibiotic with bacteriostatic activity. Similarly to the remaining tetracyclines, it inhibits the synthesis of proteins by the disruption of the 30S subunit binding.

It was developed mainly for the treatment of acne lesions. It contains a tetracyclic ring which is modified by the binding aminomethyl group at the C7 carbon [55].

Such a modification may be responsible for maintaining antibacterial activity against *Cutibacterium acnes* and decreasing the activity against the bacteria that make up the intestinal microflora [55,56]. Apart from bacteriostatic activity, it inhibits neutrophil chemotaxis and the activity of extracellular matrix metalloproteinases (see Table 2).

## 4. Adverse Effects of Tetracyclines

Side effects on the skin or interactions with other medicines.

In this article, we focus on side effects that are of particular relevance to the daily work of a dermatologist, that is, those involving the skin or interactions with other drugs.

### 4.1. Phototoxicity

Tetracyclines usually trigger phototoxicity, but not photoallergy. The former is independent from the immune response, so it may also occur during the first exposure to the drug. However, due to the dose-dependence phenomenon, it occurs when the suitable amount of the drug is concentrated in the skin and it is exposed to suitable quantities of non-ionizing electromagnetic radiation.

UVA (320–400 nm) is the part of the solar spectrum which is most commonly considered as being related with phototoxicity. However, UVB (290–320 nm) and visible light may contribute to the development of such a reaction [57].

Clinically speaking, a phototoxic reaction resembles a severe excessive sunburn characterized by erythema, edema, occasional vesicles, and burning followed by exfoliation.

Depending on skin type and pigmentation, a reaction appears within several minutes or hours after exposure to light [58].

Older generations of tetracyclines were much more likely to trigger skin reactions. The family of tetracyclines includes numerous molecules. Not all of them induce significant phototoxic reactions. Until recently, the use of tetracyclines in summer had still been discussed. Research revealed a number of significant events ascribed to minocycline and lymecycline, while the majority of studies concerned doxycycline. There is a tendency towards the standardization of the family of tetracyclines to be defined as “photosensitizing drugs” without differentiating between various molecules.

### 4.2. Hyperpigmentation

This adverse effect is characteristic of minocycline. It is induced by the precipitation of the minocycline–iron complex in the skin [59]. The incidence of discolorations induced by minocycline ranges from 2.4% to 41% and is the highest in patients with rheumatoid arthritis. Depending on the type, discolorations are located on the face within the inflammatory areas (type 1), on the healthy skin within the forearm and shin (type 2) or diffuse discolorations which affect sun-exposed skin (type 3—the least common) [60].

### 4.3. Onycholysis, Photoonycholysis

Both onycholysis and photoonycholysis have been described in the context of tetracyclines. They can affect both hands and feet, also with some nails spared. The more frequent involvement of the fingers than the feet, which are less commonly exposed, confirms a mechanism involving UV radiation.

The first case of photoonycholysis after limecycline was described in 2014 [61].

As regards doxycycline, onycholysis of the distal part of the nail plate and nail discoloration occur at a frequency of less than 1:1000 [62].

### 4.4. Induced Lupus Erythematosus

The development of systemic lupus is an important and possible consequence of tetracycline use. It may be triggered by the use of older-generation tetracyclines, but is most commonly described in relation to minocycline.

Approximately 57 cases of minocycline-induced lupus have been fully reported to date. Arthritis was the presenting symptom in 100% of patients. It involved small and large joints of the upper and lower limbs. Skin lesions were observed in about 1/5 of patients, with the typical lupus-like butterfly-shaped erythema or discoid rash [63] affecting only one patient [64].

Tests such as antibodies to DNA, antihistones or anti-Sm antibodies seem to be less sensitive to minocycline-induced lupus.

### 4.5. Idiopathic Intracranial Hypertension

The possibility of developing increased intracranial pressure is one of the most dangerous side effects of tetracyclines. The symptom complex also described by the synonym “pseudotumor cerebri” is manifested by a headache, tinnitus synchronous with pulse and transient visual disturbances. The headache involves the frontal region, is most severe shortly after awakening and worsens when lying down [65].

According to the latest systematic review conducted in 2019, the highest category V of drugs that may cause intracranial hypertension includes tetracyclines, vitamin A and its derivatives (including isotretinoin and tretinoin), and recombinant growth hormone [66].

The pathophysiology of intracranial hypertension is not fully elucidated. It is likely that tetracyclines reduce cerebrospinal fluid outflow through the arachnoid villi, and that the metabolites of oral retinoids (retinols) have a direct toxic effect on them [67,68].

The use of tetracyclines in the treatment of infectious dermatological diseases.

In everyday dermatological practice, acne is often treated with a combination of oral antibiotics and oral retinoids. However, as tetracyclines and isotretinoin belong to the high-risk category of hypertension, concomitant use of these drugs is not recommended.

Although all tetracyclines are structurally similar, they differ significantly in their antimicrobial activity and range of side effects, which affects their clinical application.

## 5. Bacteriostatic Activity

In many dermatologic and venereal conditions, tetracyclines have been the mainstay of treatment for years because of their bacteriostatic effect. The first-line treatment for all forms of Lyme boreliosis (except late neuroboreliosis) is doxycycline at a dose of 100 mg twice a day for 14 to 28 days [69]. In early syphilis, doxycycline is an alternative treatment in case of allergy to penicillin [70]. Doxycycline given at a dose of 100 mg twice a day is the treatment of choice in nongonococcal urethritis caused by chlamydia (for 7 days) and lymphogranuloma venereum (for 21 days) [71,72]. For granuloma inguinalis, tetracyclines are a second therapeutic option and should be used for a minimum of 3 weeks or until the lesions are completely healed [73]. New and increasingly common infectious diseases of global concern caused by the group of bacteria of the genus Rickettsia are defined as spotted fevers. Most spotted fevers, which include Rocky Mountain spotted fever, Mediterranean spotted fever, Typhus group and Q fever, resolve after treatment with doxycycline for at least 10 days [74].

## 6. Non-Antibiotic Activity

The use of tetracyclines in the treatment of non-infectious dermatological diseases (see Table 3).

Based on our own experience and the latest literature, below we explain the use of tetracyclines in several skin diseases which are hard to treat.

### 6.1. Granulomatous Diseases: Necrobiosis Lipoidica, Sarcoidosis

The use of doxycycline has led to spectacular improvement in the treatment of granulomatous diseases over the past decade. Researchers published case reports concerning necrobiosis lipoidica and dermal forms of sarcoidosis [87,88].

The effectiveness of doxycycline even in the ulcerative forms of necrobiosis lipoidica is most probably associated with its anti-inflammatory activity and the inhibition of tissue remodeling through the influence on metalloproteinases [89,90,91].

Moreover, in vitro research showed that doxycycline inhibited the formation of granulation tissue via inhibiting protein kinase C [87,90].

### 6.2. Prurigo Pigmentosa

The clinical presentation of this dermatosis includes itchy brownish-erythematous papules which are mostly located in the upper part of the trunk. Reticulate hyperpigmentation is frequently observed within the exanthems. Skin lesions are accompanied by persistent pruritus [91]. The condition was first described by Nagashima in 1971 [92]. Currently, it is more and more commonly observed in patients consuming the ketogenic diet. It is often misdiagnosed as eczema. No improvement is often seen after the use of topical glucocorticosteroids. According to the authors of the case reports, immediate improvement occurred after introducing doxycycline/minocycline and resuming the consumption of complex carbohydrates, while diet modification itself brought no expected improvement [91,92,93].

### 6.3. Hidradenitis Suppurativa

Hidradenitis suppurativa/acne inversa (HS) is a chronic inflammatory skin disease characterized by painful, recurrent nodules and abscesses that rupture and lead to the formation of sinus tracts and scarring [86].

Its typical locations are body folds, most commonly the axillae, inguinal and anogenital regions, but it may affect other areas as well [94,95].

The key factor in the pathogenesis of the lesions is follicular hyperkeratosis leading to follicular occlusion. As a result of closing the follicle outlet, epidermal cells, hair elements, sebum and bacteria accumulate. Clinically, blackheads and pustules are observed at this stage, where bacterial superinfection is a secondary phenomenon. The overcrowding of the hair follicle leads to its rupture, accompanied by inflammation resembling a foreign body reaction with neutrophilic infiltration. At further stages, the contents of the follicle seek an outlet, which leads to the formation of fistulas. The healing process of such a massive inflammation leads to the fibrosis of the skin and subcutaneous tissue, and the formation of sternal scars [96,97,98].

According to European guidelines for hidradenitis suppurativa published in 2016, oral antibiotics are the first line of treatment. Tetracyclines were recommended for less severe cases (Hurley I–II) and the combination of clindamycin and rifampicin for the failure of first-line treatment or more advanced stages (Hurley II–III) [86].

In recent years, there has been much controversy regarding the long-term use of clindamycin and rifampicin. The contribution of clindamycin to therapeutic success remains questionable since rifampicin, as a known CYP3A4 inducer, was shown to reduce clindamycin blood levels by 90% [99].

It was speculated that the consequent lower clindamycin levels in combination protocols might reduce the strength of the treatment itself in cases of severe HS lesions, as draining tunnels were commonly colonized by polymorph-abundant anaerobic microflora [100].

Prolonged and not fully effective antibiotic therapy leads to the selection of resistant strains and increases the risk of adverse reactions.

Considering the pathogenesis of hidradenitis suppurativa, where bacterial superinfection is a secondary process and neutrophils are the main cells in the inflammatory infiltrate, it is worth considering the use of tetracyclines not only at the early stages. A prospective, international cohort study including 283 patients showed a similar efficacy of tetracyclines and a combination of rifampicin and clindamycin after 12 weeks of treatment, independent of the stage of the disease [101].

## 7. Conclusions

Tetracyclines are an ever-expanding group of antibiotics used in dermatology for a long time. The pleiotropic effect is responsible for the efficacy of tetracyclines in infectious diseases as well as in inflammatory dermatoses. In the near future, the planned clinical trials evaluating the therapeutic activity of tetracyclines based on their concentration in the plasma or tissues will be the basis for the rationalization of the dosage and duration of treatment in specific dermatological indications.

## Figures and Tables

**Table 1:** Characteristics of tetracycline group.

	Broad Spectrum				Narrow Spectrum	References
	1st generation		2nd generation		3rd generation	
	Tetracycline	Doxycycline	Minocycline	Lymecycline	Sarecycline	
Chemical structure	Tetracyclic naphtacene-carboxyamide ring system and conventional numbering of the condensed ring and key positions					
	methyl and hydroxyl groups at C6 carbon	hydroxyl group at C5 carbon and methyl group at C6 carbon	dimethylamine group at carbon C7	Combination tetracycline with L-lysine	aminomethyl group at the C7 carbon	[4,5,6]
Intake	mandatory meal interval	can be taken with food	can be taken with food	can be taken with food	can be taken with food	[2]

**Table 2:** The most common adverse effects of tetracycline antibiotics.

Tetracycline	Doxycycline	Lymecycline	Minocycline	Sarecycline
diarrhea increased total bilirubin levels abdominal pain vaginal candidiasis symptoms of increased intracranial pressure [40]	vaginal candidiasis nausea phototoxicity [41,43]	nausea diarrhea headache [47,49]	CNS-related manifestations (nausea, vomiting, vertigo) due to the lipophilic properties at higher doses it may cause characteristic discolorations, lupus-like lesions and irritable bowel syndrome [50,51,52]	nausea, vomiting approx. 2%, diarrhea 1% vertigo < 1% [55,56]

**Table 3:** Non-antibiotic activity of tetracyclines—use in inflammatory diseases.

	Mechanism of Action	Recommended Dosage Pattern	References
Acne	Inhibition of neutrophil chemotaxis induced by *P. acnes* Inhibition of IL-8 (a chemotactic cytokine and activator of neutrophils) Inhibition of MMP Reactive oxygen species scavenging Inhibition of phospholipase A2	Tetracycline: 1 g daily given in divided doses; when improvement occurs in 1–2 weeks, decrease slowly to a maintenance dosage of 125–500 mg daily Doxycycline: 200 mg on the first day of treatment (administered 100 mg every 12 h) followed by a maintenance dose of 100 mg/Day Minocycline: 50 mg 1–3 times daily Limecycline: 300 mg once daily for 12 weeks	[75,76,77]
Rosacea	Inhibition of the activation and degranulation of neutrophils Suppression of pro-inflammatory cytokines TNFα and IL-1β Decreasing the levels of MMP (especially MMP 9) Inhibition of the expression of nitric oxide synthase	Tetracycline 250–500 mg twice daily for 4–8 weeks p.o. Doxycycline 50–100 mg once or twice daily for 4–8 weeks p.o.	[78]
Autoimmune bullous disorder	Inhibition of MMP activity Inhibition of mast cell activation	Doxycycline 2 × 200 mg	[79]
Neutrophilic disorders: Pyoderma gangrenosum Sweet’s syndrome Neutrophilic dermatosis of the dorsal hands	Inhibition of IL-8 and neutrophil activation	1.5–2 g daily given in divided doses p.o.	[80,81]
Granulomatous diseases: Sarcoidosis Necrobiosis lipoidica	Inhibition of granuloma formation in vitro	1.5–2 g daily given in divided doses p.o.	[82]
Aphtosis, periodontitis	Inhibition of MMP	Doxycycline 20 mg p.o. Tetracycline (250 mg) used as a mouth rinse and subsequently swallowed	[83,84]
Palmoplantar pustular psoriasis	Due to limited data the exact mechanism has not been clear	Tetracycline 250 mg, twice daily p.o.	[85]
Hidradenitis suppurativa	Inhibition of proinflammatory cytokine levels Inhibition of the activation and degranulation of neutrophils Scavenging reactive oxygen species (ROS)	Tetracycline 500 mg twice daily for 4 months p.o.	[86]

## Data Availability

Not applicable.
